# The Effects of PM_2.5_ from Asian Dust Storms on Emergency Room Visits for Cardiovascular and Respiratory Diseases

**DOI:** 10.3390/ijerph14040428

**Published:** 2017-04-16

**Authors:** Ssu-Ting Liu, Chu-Yung Liao, Cheng-Yu Kuo, Hsien-Wen Kuo

**Affiliations:** 1 Institute of Environmental and Occupational Health Sciences, National Yang-Ming University, 155 Li-Nong 2nd Street, Taipei 114, Taiwan; rs86229@yahoo.com.tw; 2 Department of Early Childhood Educare, College of Health, Chung Chou University of Science and Technology, Changhua 510, Taiwan; cyliao@dragon.ccut.edu.tw; 3 College of Arts and Sciences, University of San Francisco, San Francisco, CA 94117, USA; taiwan22361397@gmail.com; 4 School of Public Health, National Defense Medical Center, Taipei 114, Taiwan

**Keywords:** air pollution, PM_2.5_, emergency room, Asian dust storms, case-crossover

## Abstract

A case-crossover study examined how PM_2.5_ from Asian Dust Storms (ADS) affects the number of emergency room (ER) admissions for cardiovascular diseases (CVDs) and respiratory diseases (RDs). Our data indicated that PM_2.5_ concentration from ADS was highly correlated with ER visits for CVDs and RDs. The odds ratios (OR) increased by 2.92 (95% CI: 1.22–5.08) and 1.86 (95% CI: 1.30–2.91) per 10 µg/m^3^ increase in PM_2.5_ levels, for CVDs and RDs, respectively. A 10 µg/m^3^ increase in PM_2.5_ from ADSs was significantly associated with an increase in ER visits for CVDs among those 65 years of age and older (an increase of 2.77 in OR) and for females (an increase of 3.09 in OR). In contrast, PM_2.5_ levels had a significant impact on RD ER visits among those under 65 years of age (OR = 1.77). The risk of ER visits for CVDs increased on the day when the ADS occurred in Taiwan and the day after (lag 0 and lag 1); the corresponding risk increase for RDs only increased on the fifth day after the ADS (lag 5). In Taiwan’s late winter and spring, the severity of ER visits for CVDs and RDs increases. Environmental protection agencies should employ an early warning system for ADS to reduce high-risk groups’ exposure to PM_2.5_.

## 1. Introduction

Asian dust storms (ADS) frequently occur in northern China in late winter and spring (from November to May). Asian dust follows westerly winds that move toward the east, impacting Japan, Korea, and other neighboring areas. Only under specific meteorological conditions will an ADS impact Taiwan. On 25 April 2009, ADS arrived in Taiwan, bringing with it PM_10_ (particles are represented by the mass concentration of particles smaller than 10 μm) exceeding 1000 µg/m^3^. This was the most serious case in Taiwan. Since then, the situation has worsened, along with the desertification of China’s Inner Mongolia, a global increase in droughts, and the uneven distribution of rain. These increases in both the frequency and severity of ADS have impacted Taiwan [1], worsening air quality and visibility. The increase in the number of particulates floating in the air has led to an increase a number of problems, including allergic nasal inflammation, asthma, eye problems, skin allergies, and skin irritability. Asian Dust Storms in Taiwan have led to an increase of 7.66% in the risk for respiratory diseases (RDs) one day after the ADS (lag 1), 4.92% in total deaths two days after the ADS (lag 2), and 2.59% in circulatory diseases two days after the ADS (lag 2). None of these effects were statistically significant [2]. However, there is a statistically significant association between ADS events and daily primary intracerebral hemorrhagic stroke visits three days after the event (relative risk of 1.15; 95% CI: 1.01–10.10) [3].

Previous environmentally epidemiological studies have shown positive correlations of particulate matter (PM) on the risk of death [4,5,6,7,8,9], hospitalizations [3,10,11,12,13,14,15,16,17], and emergency room (ER) visits [8,18,19,20,21]. In Barcelona, Spain, a case-crossover study from 2003 to 2007 indicated that PM_2.5_ from car exhaust, gas combustion, hyponitrites, hydrosulfites, and road dust are correlated to CVD mortality (*p* < 0.05); for every 10μg/m^3^ increase in PM_2.5_ (the mass concentration of particles smaller than 2.5 µm), cardiovascular-related mortality increased by 2.9% (95% CI: 1.4–4.4) [9]. Previous research on PM has focused on its effects on deaths or hospital visits due to diseases. Few studies have investigated the effects of ADS on ER visits. In addition, the majority of previous ADSs studies used PM_10_ as the exposure index. However, recent epidemiological studies [22,23] have found PM_2.5_ to pose a larger health threat due to its smaller size and larger surface area, which allows it to carry more toxic substances. Hence, the World Health Organization (WHO) has already recommended the replacement of PM_10_ with PM_2.5_ for studies on air quality index [24]. Following that recommendation, Taiwan’s EPA began measuring PM_2.5_ levels at its environmental monitoring stations and performing research on the health impacts of PM_2.5_’s. Due to Taiwan’s small geographic area and its intermixing of traffic pollutants with industry pollutants, the PM_2.5_ composition might differ from that in the United States or Europe [25,26]. Thus, the PM_2.5_ of other countries might not be the same as Taiwanese PM_2.5_. Whereas the characteristics of PM_2.5_ in others countries might not represent exposure to PM_2.5_ in Taiwan, we performed a case-crossover design assessing the PM_2.5_ of ADS. This investigation particularly focused on the effects of PM_2.5_ has on the risk of ER visits for CVDs and RDs in central Taiwan.

## 2. Materials and Methods

### 2.1. Study Design

The case-crossover design is based on one proposed by Maclure in 1991 [27] to investigate the health effects of short-term exposure to hazards; it is a form of observational study design. This method has already been applied in the literature on the short-term effects of air pollution on health. In this design, each participant is compared to himself before or after the exposure. Because this design compares each participant to himself, it relieves researchers of the need to find a control. In addition, it allows for the control of each individual confounder. For these reasons, we chose the unidirectional case-crossover design for our study. In our study, the day of ER visits acted as the exposure period, while 14 days prior acted as the control period. To estimate relative risk, the exposure frequency during ADS periods just before outcome onset is compared with exposure frequencies during control times.

### 2.2. ER Visits Data

We used a National Health Insurance (NHI) database in central Taiwan as our dataset. NHI began in 1995, and as of 2013 has a coverage rate of 99.9%. It is thus representative of the entire Taiwanese population and its medical institutions. Demographic information in the registry of ED visit included gender, age and delivering time and diagnosis of diseases, etc. No other detailed personal information was recorded. According to the International Classification of Diseases, 9th Revision (ICD-9), we filtered the dataset to extract those with CVDs (ICD-9 codes 90-459) and those with RDs (ICD-9 codes 460-519). The study was conducted in accordance with the Declaration of Helsinki, and the protocol was approved by the Ethics Committee of the Institutional Review Board (IRB) of National Yang-Ming University (Project identification code: YM102007E).

### 2.3. Exposure Data and Meteorological Data

The so-called “Asian Dust Storm” (ADS) is a strong wind that collects large amounts of dust from the ground, creating a weather condition that negatively affects visibility. Generally, it is a result of drought and desertification. Meteorologists define ADS which the visibility is below 1 km and levels of PM_10_ exceeded 150 µg/m^3^ in consecutive three-days period [1]. Our study relied on the data from 2006 to 2008 from nine Taiwan EPA air-quality monitoring stations, which fully cover cases’ residential address. As per the Taiwanese EPA’s definition of an ADS, we determined the presence of ADS if two conditions were met: a visibility of less than 1 kilometer and a PM_10_ level of over 150 µg/m^3^. With this definition, we selected 16 ADSs that occurred between 2006 and 2008, six events in 2006, eight in 2007, and two in 2008. Monitoring stations were fully automated and provided daily readings of sulfur dioxide (SO_2_) (by ultraviolet fluorescence), PM_10_ (by β-ray absorption), nitrogen dioxide (NO_2_) (by ultraviolet fluorescence), carbon monoxide (CO) (by non-dispersive infrared photometry), and ozone (O_3_) (by ultraviolet photometry) levels. Daily meteorological information, including the mean temperature and mean humidity, was provided by the Central Weather Bureau. The monitoring of PM_2.5_ was done by tapered element oscillating microbalance (TEOM), which began in 2006. TEOM allowed for two types: manual monitoring and automatic monitoring. As per the standard measurement guidelines for measuring air quality in Taiwan, we used manual monitoring to collect data on PM_2.5_. To ensure consistency between the manual and automatic monitoring styles, we referenced the American EPA methodology, which suggests the application of simple linear regression on the manual and automatic data, allowing for the correction of the automatic data and immediate reporting of changes as a warning system. We calculated the average of the data in each 24-h period for each of the nine monitoring stations in central Taiwan, and then this data combined with ER visits databases.

### 2.4. Statistics Analysis

We began by organizing the data in Microsoft Excel 2013. Using SAS 9.3, we ran conditional logistic regression comparisons on the exposure and control periods. To understand the effects of short-term PM_2.5_ exposure on CVDs and RDs in ER visits, we estimated the odds ratios (OR) and took their 95% confidence intervals (CI). Because the temperature changes between the seasons in Taiwan are not significant, we ignored the interaction effects of PM_2.5_ and season on CVDs and RDs. In consideration of age and sex effects, we added an age variable (≥65 or <65 years of age) and a sex variable. In addition, due to individual differences in the physical reaction to PM_2.5_ exposure, we added a lag effect variable (lag 0 to lag 5). A lag effect of zero (lag 0) corresponds to the same day of exposure to PM_2.5_—that is, the day of arrival at the hospital for ER visits, which means the day of the individual CVDs or RDs event (depending on the time scale being studied) was the initial exposure period (lag 0) considered for that case. For the daily time scale analysis, we examined the association at the day of onset (lag 0 day) and the 1 to 5 before onset (lag 1, 2, 3, 4, 5). Single-pollution model, the two-pollution model, and the multi-pollution model were used in our analysis of PM_2.5_ and other pollutants (SO_2_, CO, O_3_, NO_2_, NO and NOx) effects on the ER visits for CVDs and RDs.

## 3. Results

Table 1 indicates significant differences for PM_10_, PM_2.5_, SO_2_, O_3_, average daily temperature (ADT), DTR (diurnal temperature range), and relative humidity (RH) between ADS days (index days) and comparison days. During ADS periods, PM_10_, PM_2.5_, SO_2_ and O_3_ were higher in concentration on index days. However, the average temperature, relative humidity, NO, and NO_x_ were lower. We found no significant differences in the levels of CO and NO_2_ across index days and comparison days.

Table 2 uses Pearson’s correlation coefficients to assess the correlations among different air pollutants. We found positive correlations between PM_10_ and PM_2.5_ (*r* = 0.76), SO_2_ (*r* = 0.51), as well as CO (*r* = 0.34); we found negative correlations between PM_10_ and RH (*r* = −0.34). PM_2.5_ had positive correlations with SO_2_ and CO (*r* = 0.45 and *r* = 0.54, respectively). SO_2_ was positively correlated with CO, NO and NO_x_, with correlation coefficients of 0.55, 0.36 and 0.28; it had negative correlations with O_3_, ADT and RH (*r* = −0.32, *r* = −0.31 and *r* = −0.48). O_3_ had negative correlations with CO (*r* = −0.63), NO_2_ (*r* = −0.75), NO (*r* = −0.69), and NO_x_ (*r* = −0.74). ADT was positively correlated with RH (*r* = 0.66), though DTR was negatively correlated with RH (*r* = −0.48).

Table 3 shows the effects of PM_2.5_ on ER visits for the susceptible groups with CVDs and RDs. For those 65 years of age, a 10 μg/m^3^ increase in PM_2.5_ led to a 2.77-fold (*p* < 0.05) increase in the risk of ER visits for those with CVDs. Likewise, we found a 3.94-fold increase in the risk of ER visits for those with RDs. For those <65 years of age, a 10 μg/m^3^ increase in PM_2.5_ led to OR of 1.77 (*p* < 0.05) for the risk of ER visits for RDs. A 10 μg/m^3^ increase in PM_2.5_ for men, OR was 1.80 (*p* < 0.05) for the risk of ER visits for RDs, and OR was 1.99 for the risk of ER visits for CVDs. We found significant effects on women whose ORs were 3.09 and 1.86 for ER visits for CVDs and RDs, respectively.

Table 4 shows the lag effect of PM_2.5_ on CVD and RD emergency visits. During the first and second day of the sand-dust storm arriving in Taiwan (lag 0 and lag 1, respectively), the increase in PM_2.5_ seems to have led to an increase in the ER visits for CVDs. As for the exposure period’s effect on ER visits for RDs, every day (lag 0 to lag 5) besides lag 3 had a significant effect.

Table 5 shows the three models’ (single-, two- and multi- pollutant) descriptions of the effects of the heightened PM_2.5_ during the exposure period on ER visits for CVDs and RDs. In the single-pollutant model, which does not account for other pollutants, a 10 μg/m^3^ increase in PM_2.5_ levels led to an OR of 2.20 (95% CI: 1.22–5.08) for ER visits for CVDs and an OR of 1.86 (95% CI: 1.30–2.91) for ER visits for RDs. For the two-pollutant model, the models that added NO_2_, CO, NO or NO_x_ showed a significant correlation between PM_2.5_ and ER visits for CVDs; models that added SO_2_, NO_2_, O_3_, CO, NO or NO_x_ showed a significant correlation between PM_2.5_ and ER visits for RDs. In the multi-pollutant model, PM_2.5_ showed no statistical correlation with ER visits for CVDs, but we found statistical significant correlations with RDs in the models including SO_2_ + NO_2_, SO_2_ + CO_2_, SO_2_ + NO_x_, NO_2_ + CO, O_3_ + NO_x_ and CO + NO_x_.

## 4. Discussion

The desertification of northern China has worsened in recent years, which, when combined with the global increase of drought and uneven distribution of rainfall, has led to a rise in both the frequency and severity of Asian Dust Storms. Moreover, ADSs differ from other dust storms: First, the particulates of ADS reaching Taiwan are very small. This is because the long distance the ADS must travel prevents the transportation of larger particles; gravity eventually brings those larger particles back to the ground or sea. As a result, the particulate size ranges from 1.35~10 μm. Second, the average concentration of air pollutants during ADS is significantly high mainly because ADS form as clouds of dust, not as high-speed winds that carry dust with them. For this reason, ADS tend to carry with them a larger amount of toxins [23,24]. Because the voyage of ADS is affected by dissipation, subsidence, and filtering due to rainfall, the Taiwanese EPA uses meteorological observatory data and other countries’ dust storm models to track ADS, confirming whether they will impact Taiwan. After a detailed analysis that predicts a decrease in Taiwan’s air quality as a result of the ADSs, the EPA will issue a warning to the citizens of Taiwan, encouraging them to take preventative measures against the negative effects of ADS. The observation of the ADS continues until it leaves Taiwan. An ADS increases the concentrations of PM_2.5_ in central Taiwan. During an ADS, the PM_2.5_ level is 61.93 μg/m^3^, while that on comparison days is 48.87 μg/m^3^. Our findings are the same as those of Kamouchi et al. [28] and Kwon et al. [29]. Yet the levels of combustion particulates (CO and NO_2_) do not differ during an ADS, a result that matches that of Yang et al. [30].

When attempting to understand the effects of air pollutants on daily emergency visits, deaths, and hospital visits, we must also control for seasonal and other time variables. Our study design employed a unidirectional recall case-crossover methodology instead of a bidirectional case-crossover design. Using the time point of 14 days prior to the sand-dust storm as our control, we were able to control confounders for the long-term and for different season. In addition, by having looked to the past, we could control for the risks of physical pain that occur after emergency visits and exposure to PM_2.5_. Through this case-crossover design, we were able to control for many confounders without having to rely on statistical models. However, some research has shown that unidirectional case-crossover study designs can overestimate odds ratios. Because ER visits and air pollution are both susceptible to time trends, when looking at exposure intensity, the control period could be systematically higher or lower than the exposure period [15].

Our study makes a contribution to research on the correlation between PM_2.5_ from ADS and CVDs from RDs ER visits. Compared to other studies, our study found a larger effect when PM_2.5_ levels increased every 10 μg/m^3^. One possibility for this is that the PM_2.5_ of our research could have a different chemical composition [23]. Principal component analysis (PCA) was used to analyze the components of Taiwanese ADS and found three principle components were able to explain 88% of the variation. The first component (PC1) explained 52% of the variation and represented a larger-than-average concentration of NO and NO_x_ (factor loading >0.70). These components mainly came from industry and traffic exhaust. The second component (PC2) explained 24% of the variation and consisted of PM_2.5_, SO_4_^2−^, NO_3_^−^, OC, as well as highly dense mineral components and pollutions such as sulfate and nitrate. These components mainly came from Chinese factories [31]. The main component in the ADSs of 2002 in Taiwan was sulfate, as ADSs do no only carry dust but also combustion by-products [32].

Our study shows that, according to the single-pollutant model (i.e., a model that does not adjust for the effects of other pollutants), a 10 μg/m^3^ increase in PM_2.5_ corresponds with statistical significance to an increase in CVD and RD emergency room visits. For the two-pollutant models that adjusted for NO_2_, CO, NO and NO_x_, a 10 μg/m^3^ increase in PM_2.5_ led to an increase in ER visits for CVDs in these models that controlled for CO showed the largest effect. In the two-pollutant models adjusted for SO_2_, NO_2_, O_3_, CO, NO and NO_x_, a 10 μg/m^3^ increase in PM_2.5_ led to more RD emergency visits. Again, that controlling for CO showed the largest effect. Other research [33] in Taiwan has pointed out that despite other air pollutants having an effect on ER visits (e.g., NO_2_, CO and O_3_ increasing visits for RDs as well as CO, NO_2_ and SO_2_ increasing ER visits for heart diseases), the relative risks of these pollutants is less than that of PM_2.5_. In multi-pollutant models, adjusting for CO and NO_2_ still results in the relative risk (RR) for PM_2.5_ being higher. We created cut-off points for the groups most easily affected by PM_2.5_ and discovered that people over the age of 65 or women were more likely to ER visits during the extra PM_2.5_ exposure that ADSs brought. Seniors typically have higher ER visits for CVD, which leads to a higher susceptibility to PM_2.5_. Other research [17] has supported this fact by showing that those under the age of 65 are less likely than their older counterparts to be hospitalized for CVDs during PM_2.5_ exposure. Women might be more susceptible because of hormones, but research on the interaction between sex and air pollution has not yet proved such a thing [34]. ER visits for respiratory problems occurs more often in the <65 years of age, and sex differences in this case do not differ significantly in the OR. Nevertheless, regardless of sex, increased PM_2.5_ exposure increases the risk of respiratory problems. Other research [14] on why those under the age of 65 are more likely to treatment for respiratory problems during an ADS has investigated outside activity time and immune system mechanisms, citing them as probable factors.

Evidence has shown that exposure to air pollution can have an adverse effect on the protective abilities of the lungs, such as the dynamic filtering of air, mucociliary cleaning, particulate dispersion, and alveolar macrophage expulsion. Macrophage can inhibit the propagation of viruses, destroying fragmented cells and restricting the transmission of the virus through the production of antigens for T lymph cells. Exposure to PM_2.5_ can lead to an inflammatory reaction in the lungs, exacerbating lung diseases. It can also release harmful enzymes in the cell as well as alter other reactions related to blood coagulation and its triggering. Consequences of this include cardiovascular problems that require urgent care and the development of atherosclerosis [35]. Furthermore, air particulate pollution significantly correlated with autonomic function measured by changes in heart rate variability (HRV) and blood markers of inflammation. The result is an increase in heart rate and the variance of that speed [13]. Because PM_2.5_ acts on different disease mechanisms for CVDs and RDs, we used a lag effect to investigate the differences in timing when ADS’s PM_2.5_ levels increases affects these two diseases. As PM_2.5_ levels increases led to more emergency visits for CVDs on the day of the ADS (lag 0) and on the day after (lag 1), we conclude that exposure to PM_2.5_ during a ADS has a more urgent effect on CVDs, a result that matches that of Kamouchi et al. [25]. Exposure to PM_2.5_ brought by ADS affects RDs over the course of all five days, with only lag 3 not having statistical significance. This result shows that the exposure to PM_2.5_ from ADS does not have such an immediate effect on RDs as it does on CVDs. The reason for this is that the PM_2.5_ in ADS differs from microorganisms. A microorganism must attach to the sand and dust, then enter the body, where they can finally do harm to the respiratory system [25].

Our study has some limitations. First, we used outdoor monitoring stations to collect the average daily air pollution levels and other weather variables as our representatives for human exposure to air pollutants. However, other factors—such as the use of air conditioning and the ventilation between indoors and outdoors—might influence how representative our sample truly is, thereby affecting our results and their implications [34]. Second, our study from NHI data did not include individual behavioral data. Other studies have shown how heavy smokers or drinkers are more susceptible to the negative health effects of ADSs [25]. Third, we assumed that PM_2.5_ to be located in specific areas and that our designated areas were homogenous in airborne quality. We determined that these areas can represent the average air quality situation in central Taiwan as pertains to PM_2.5_. However, we were without data on the components of PM_2.5_ and therefore could not establish exactly what part of PM_2.5_ is responsible for the negative health effects we found. Hence, during an ADS, the concentrations and types of suspended fine particles could have different levels of negative effects on health. From a study in Beijing [36], the PM_2.5_ concentration was approximately 230 μg/m^3^ and the crustal elements constituted about 66.4% of the chemical composition of PM_2.5_ while sulfate and nitrate contributed much less during the ADS period. We therefore can only suggest that susceptible groups stay indoors during an ADS. However, the doors and windows of a household can only offer limited protection, and the quality as well as the maintenance of the air conditioning systems might be lacking. PM_2.5_ can easily enter households, carrying along with them pollutants [37]. Thus, environmental protection organizations should not only implement measures to offer earlier warnings of ADSs but should also advise at-risk groups (asthmatics, those with CVD or RD, people with allergies—and especially the elderly as well as young children) to remain indoors. These organizations should also emphasize that people check the sealing quality on door and screens.

## 5. Conclusions

This study has shown that exposure to high levels of PM_2.5_ as brought by ADSs increases the risk of ER visits for CVDs and RDs, especially in people above the age of 65 or women. However, those under the age of 65 are also affected by the increase in PM_2.5_ brought by ADSs in that they are more likely to require ER visits for RDs. For this reason, Asian nations should track the ADSs arriving from China as part of a precautionary system that can ensure their citizens’ health.

## Figures and Tables

**Table 1:** Comparison of various pollutants and metrological data between index and comparisons days.

Variable	Index Days (Mean ± SD)	Comparison Days (Mean ± SD)	*p*-Value
PM_10_ (μg/m^3^)	133.02 ± 31.62	77.77 ± 31.48	<0.001
PM_2.5_ (μg/m^3^)	61.93 ± 17.11	48.87 ± 22.34	<0.001
SO_2_ (ppb)	7.16 ± 2.45	5.66 ± 2.78	<0.001
CO (ppm)	0.63 ± 0.12	0.65 ± 0.21	0.425
O_3_ (ppb)	32.44 ± 9.90	23.31 ± 8.65	<0.001
NO (ppb)	6.45 ± 4.59	9.69 ± 7.28	<0.001
NO_2_ (ppb)	25.14 ± 8.21	25.28 ± 8.53	0.881
NO_x_ (ppb)	31.50 ± 12.52	34.93 ± 14.65	0.021
ADT (°C)	18.91 ± 3.84	21.25 ± 3.84	<0.001
DTR (°C)	6.67 ± 2.02	6.15 ± 1.67	0.030
RH (%)	56.37 ± 12.14	70.63 ± 4.56	<0.001

**Table 2:** Correlations between concentrations of pollutants and meteorology on Asian Dust Storms (ADS) periods.

Variable	PM_2.5_	SO_2_	O_3_	CO	NO_2_	NO	NO_x_	ADT	DTR	RH
PM_10_	0.76 **	0.51 **	0.01	0.34 *	−0.03	−0.11	−0.06	0.10	0.11	−0.34 *
PM_2.5_		0.45 *	−0.04	0.54 **	−0.07	−0.15	−0.10	0.22	−0.15	−0.02
SO_2_			−0.32 *	0.55 **	0.22	0.36 *	0.28 *	−0.31 *	0.05	−0.48 **
O_3_				−0.63 **	−0.75 **	−0.69 **	−0.74 **	−0.09	0.16	−0.20
CO					0.57 **	0.46 *	0.55 **	0.38 *	−0.09	0.28 *
NO_2_						0.92 **	0.99 **	0.32 *	0.28 *	0.16
NO							0.97 **	0.05	0.31 *	−0.02
NO_x_								0.23	0.30 *	0.10
ADT									−0.12	0.66 **
DTR										−0.48 **

* *p* < 0.05, ** *p* < 0.0001.

**Table 3:** A 10 μg/m^3^ increase in PM_2.5_ during ADS correlated with cardiovascular and respiratory diseases in emergency room (ER) based on age and sex.

Variable	Cardiovascular Diseases			Respiratory Diseases		
	OR	95% CI		OR	95% CI	
≥65	2.77 *	1.01	26.69	3.94	0.75	NA
<65	1.92	0.95	5.31	1.77 *	1.24	2.79
Male	1.99	0.80	8.77	1.80 *	1.13	3.58
Female	3.09 *	1.15	26.80	1.86 *	1.15	3.45

* *p* < 0.05.

**Table 4:** Lag effects for PM_2.5_ during ADS correlated with cardiovascular diseases (CVDs) and respiratory diseases (RDs) in the single-pollutant model.

Lag	Cardiovascular Diseases			Respiratory Diseases		
	OR	95% CI		OR	95% CI	
Lag 0	2.20 *	1.22	5.08	1.86 *	1.30	2.91
Lag 1	1.77 *	1.05	3.97	1.48 *	1.03	2.31
Lag 2	1.86	0.78	5.94	3.78 *	1.84	10.55
Lag 3	0.91	0.652	1.23	1.24	0.95	1.62
Lag 4	0.82	0.60	1.11	1.20 *	1.01	1.41
Lag 5	1.79	0.94	3.43	1.54 *	1.14	2.07

* *p* < 0.05.

**Table 5:** Odds ratio (OR) of disease associated with 10 μg/m^3^ increase of PM_2.5_ with single- and multiple-pollutant models.

Pollutant	Cardiovascular Diseases			Respiratory Diseases		
	OR	95% CI		OR	95% CI	
Single pollutants models						
PM_2.5_	2.20 *	1.22	5.08	1.86 *	1.30	2.91
Two pollutant model						
+SO_2_	7.29	0.95	55.96	2.66 *	1.18	6.00
+NO_2_	2.24 *	1.06	4.74	2.20 *	1.35	3.59
+O_3_	1.70	0.86	3.36	1.60 *	1.03	2.48
+CO	4.39 *	1.10	17.55	5.43 *	1.58	18.64
+NO	3.15 *	1.16	8.57	2.97 *	1.12	7.90
+NO_x_	2.60 *	1.18	5.77	2.51 *	1.33	4.76
Three pollutant model						
+SO_2_ + NO_2_	9.24	0.91	94.27	2.52 *	1.26	5.03
+SO_2_ + O_3_	4.08	0.47	35.03	1.74	0.91	3.32
+SO_2_ + CO	7.32	0.88	61.18	5.24 *	1.45	18.94
+SO_2_ + NO	5.59	0.41	76.68	2.89	0.74	11.32
+SO_2_ + NO_x_	5.94	0.76	46.29	2.51 *	1.12	5.65
+NO_2_ + O_3_	NA	NA	NA	1.63	0.96	2.78
+NO_2_ + CO	11.16	0.56	222.27	4.73 *	1.54	14.52
+NO_2_ + NO	NA	NA	NA	3.61	0.98	13.35
+NO_2_ + NO_x_	NA	NA	NA	3.59	0.96	13.34
+O_3_ + CO	2.51	0.34	18.57	4.46	0.95	20.87
+O_3_ + NO	3.07	0.53	17.65	NA	NA	NA
+O_3_ + NO_x_	1.32	0.59	2.97	2.96 *	1.13	7.78
+CO + NO	2.42	0.48	12.20	2.27	0.84	6.17
+CO + NO_x_	6.53	0.88	48.34	3.50 *	1.26	9.73
+NO + NO_x_	NA	NA	NA	3.62	0.98	13.36

* *p* < 0.05, NA: non-available.

## Abbreviations

ADS	Asian dust storm
EPA	Taiwan Environmental Protection Administration
TEOM	Tapered Element Oscillating Microbalance
CI	Confidence interval
ER	Emergency room
ICD-9	International Classification of Diseases, 9th Revision
NO_2_	Nitrogen dioxide
NO_x_	Nitrogen oxides
PM_2.5_	Fine particulate matters with an aerodynamic diameter of less than 2.5 μm
SO_2_	Sulfur dioxide
CO	Carbon monoxide
O_3_	Ozone
ADT	Average daily temperature
DTR	Diurnal temperature range
RH	Relative humidity
CVD	Cardiovascular diseases
RD	Respiratory diseases
WHO	World Health organization
NHI	National Health Insurance
OR	Odds ratios
